# Single nucleotide polymorphisms affect RNA-protein interactions at a distance through modulation of RNA secondary structures

**DOI:** 10.1371/journal.pcbi.1007852

**Published:** 2020-05-07

**Authors:** Elan Shatoff, Ralf Bundschuh

**Affiliations:** 1 Department of Physics, The Ohio State University, Columbus, Ohio, United States of America; 2 Center for RNA Biology, The Ohio State University, Columbus, Ohio, United States of America; 3 Department of Chemistry and Biochemistry, The Ohio State University, Columbus, Ohio, United States of America; 4 Division of Hematology, Department of Internal Medicine, The Ohio State University, Columbus, Ohio, United States of America; New York University, UNITED STATES

## Abstract

Single nucleotide polymorphisms are widely associated with disease, but the ways in which they cause altered phenotypes are often unclear, especially when they appear in non-coding regions. One way in which non-coding polymorphisms could cause disease is by affecting crucial RNA-protein interactions. While it is clear that changing a protein binding motif will alter protein binding, it has been shown that single nucleotide polymorphisms can affect RNA secondary structure, and here we show that single nucleotide polymorphisms can affect RNA-protein interactions from outside binding motifs through altered RNA secondary structure. By using a modified version of the Vienna Package and PAR-CLIP data for HuR (ELAVL1) in humans we characterize the genome-wide effect of single nucleotide polymorphisms on HuR binding and show that they can have a many-fold effect on the affinity of HuR binding to RNA transcripts from tens of bases away. We also find some evidence that the effect of single nucleotide polymorphisms on protein binding might be under selection, with the non-reference alleles tending to make it harder for a protein to bind.

## Author summary

Single nucleotide polymorphisms are single nucleotides in a genome that vary between subsets of a population. They are known to cause many diseases, but the ways in which they cause disease are often unknown. We show that one way in which single nucleotide polymorphisms may cause disease is by altering how RNA molecules fold. The nucleotides in an RNA molecule can base pair to each other to form complicated structures. This structure dictates where some proteins can bind, since many proteins can only bind to single stranded RNA. If the change from one base to another base caused by a single nucleotide polymorphism causes a protein binding motif to become preferentially base paired, then it will be harder for a protein to bind there. By modelling the folding of RNAs in conjunction with protein binding *in silico*, we show that single nucleotide polymorphisms can affect the ability of specific proteins to bind to RNAs, usually making it harder for proteins to bind.

## Introduction

Single Nucleotide Polymorphisms (SNPs) and Single Nucleotide Variations (SNVs) are nucleotide changes at single genomic positions that differ between significant subsets of a population, or general mutations that often arise due to diseases such as cancer, respectively [1]. While very common and known to cause many diseases, their effects on gene expression, protein binding, and ways in which they cause disease are not completely understood [2]. Missense mutations in coding regions are easily linked to disease, since they cause translation of a defective protein [3], but most SNPs (∼93% of disease and trait associated SNPs in genome-wide association studies) occur in non-coding regions [4]. Non-coding SNPs can appear in non-coding RNAs, introns, or in 5’ and 3’ untranslated regions (UTRs). Because these non-coding SNPs do not produce an altered protein, the pathways through which they cause disease are less well known, but they are still regularly associated with disease [5]. Understanding the effect of these non-coding or same-sense SNPs has wide-ranging implications for understanding disease, as well as evolutionary genetics [6, 7].

A possible explanation of the effect on phenotype of SNPs in 5’ and 3’ UTRs or non-coding RNAs is that they affect crucial interactions between an RNA and other biomolecules. Indeed, RNAs naturally interact with RNA-binding proteins (RBPs), RNA-protein complexes like the ribosome and the spliceosome, as well as with other RNAs [8–10]. These interactions control every step in an RNA’s life cycle, such as the life time of an RNA molecule, its subcellular localization, and the recruitment of ribosomes to mRNA molecules and ultimately the amount of protein expressed per transcribed mRNA [11, 12]. Thus, it is not surprising that interrupting these interactions is known to cause disease [13]. In line with their importance, there are over 1500 RNA binding proteins and thousands of microRNAs annotated in the human genome alone [14, 15].

It is clear that a SNP will affect protein or microRNA binding if it occurs directly on a binding site [16, 17]. However, as we will show, SNPs are also able to affect protein (or microRNA) binding “at a distance” through the involvement of RNA secondary structure. RNA secondary structures form due to the propensity of the nucleotides of an RNA to base pair [18]. For structural RNAs these base pairings are a significant determinant of the functionally relevant physical shape of the RNA, but messenger and non-coding RNAs that are not necessarily designed for specific structures will also form base pairs and thus secondary structure [19]. As microRNAs and a large fraction of RNA binding proteins bind to unpaired bases only, RNA secondary structure competes with binding of microRNAs or single-stranded RNA binding proteins and thus affects the binding affinity of the RNA for these molecules. For example, we have previously shown the existence of secondary structure mediated cooperativity between RNA binding proteins: binding of one protein to an RNA changes the ensemble of possible secondary structures by excluding the bases in its footprint from base-pairing [20, 21]. This change in secondary structures modifies the accessibility of the footprint for a second protein and thus the affinity of the RNA for this second protein. Depending on the specific sequence one binding event can make the other binding event easier or harder.

It has also been shown experimentally that specific SNPs can affect the secondary structures of mRNAs [22], and that SNPs can cause disease through changes in RNA secondary structure [23–25]. Here, we show how single nucleotide changes in an RNA molecule can, by making different conformations energetically more or less favorable, also change secondary structure drastically enough to change the affinity of an RNA for an RNA binding protein or a microRNA, and that there is some evidence that this effect might be under selective pressure in the human transcriptome. For simplicity, in the rest of the paper we will refer to the molecules binding to RNAs as “proteins”, even though these binding events could equally occur with mircoRNAs, as shown in [26], or any other molecule that binds single-stranded RNA. Likewise, we will be referring to the effect of “SNPs” on RNA-protein binding, but these effects should occur equally with any point mutation including SNVs. By computationally folding RNAs using a modified version of the Vienna RNA Package, we are able to quantitatively measure the effect of SNPs on protein binding. Using known human SNPs and PAR-CLIP data, we investigate the genome wide effect of SNPs on HuR (ELAVL1) binding. HuR is an extensively studied RNA binding protein with nearly 500 articles on PubMed. It is a member of the ELAVL family of RNA-binding proteins that selectively bind AU rich sequences, and HuR binds with a 7 nucleotide footprint mostly in the UTRs of many mRNAs [27]. HuR has diverse functions, including stabilizing mRNAs against degradation as a means of regulating gene expression and controlling nuclear export of mRNAs, and has been implicated in several diseases including cancer [28, 29]. We find that SNPs can have a many-fold effect on the binding affinity of HuR binding to RNA transcripts from tens of bases away, simply through changes in secondary structure, and propose this as a general mechanism through which SNPs can affect protein binding.

## Results

### Sequence alterations affect RNA-protein binding at a distance through changes in secondary structure

**Fig 1:**
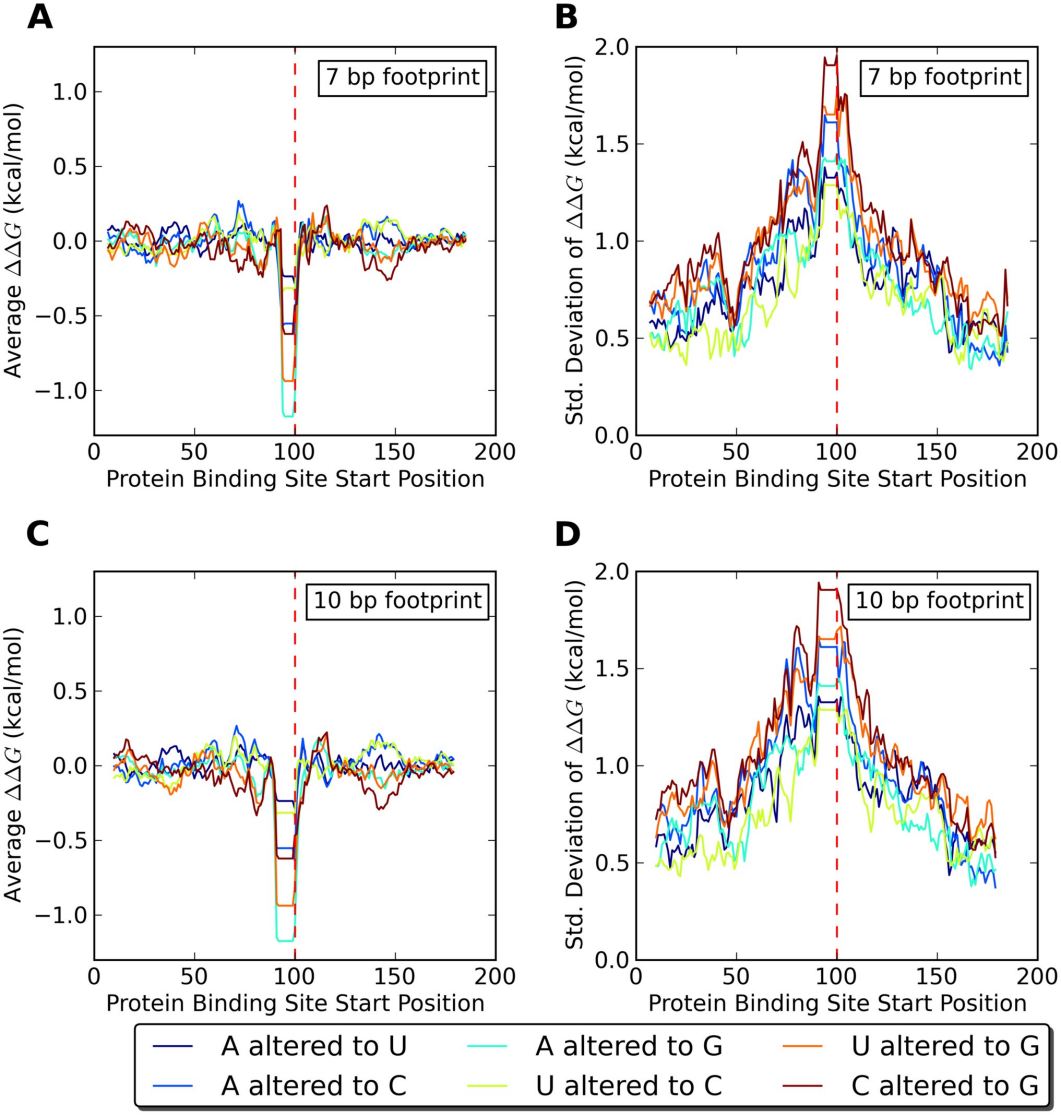
Effect of SNPs in random 201 nucleotide sequences on protein binding. (A) and (C) Averages and (B) and (D) standard deviations for the change in effective RNA-protein binding free energy, ΔΔ*G*, in response to six different single nucleotide sequence alterations averaged over 100 randomly chosen RNA sequences. (A) and (B) show data for a protein with a 7 bp footprint and (C) and (D) for a protein with a 10 bp footprint. The sequence alteration location (indicated by the dashed vertical red line) is static while the protein binding site start position is variable.

**Fig 2:**
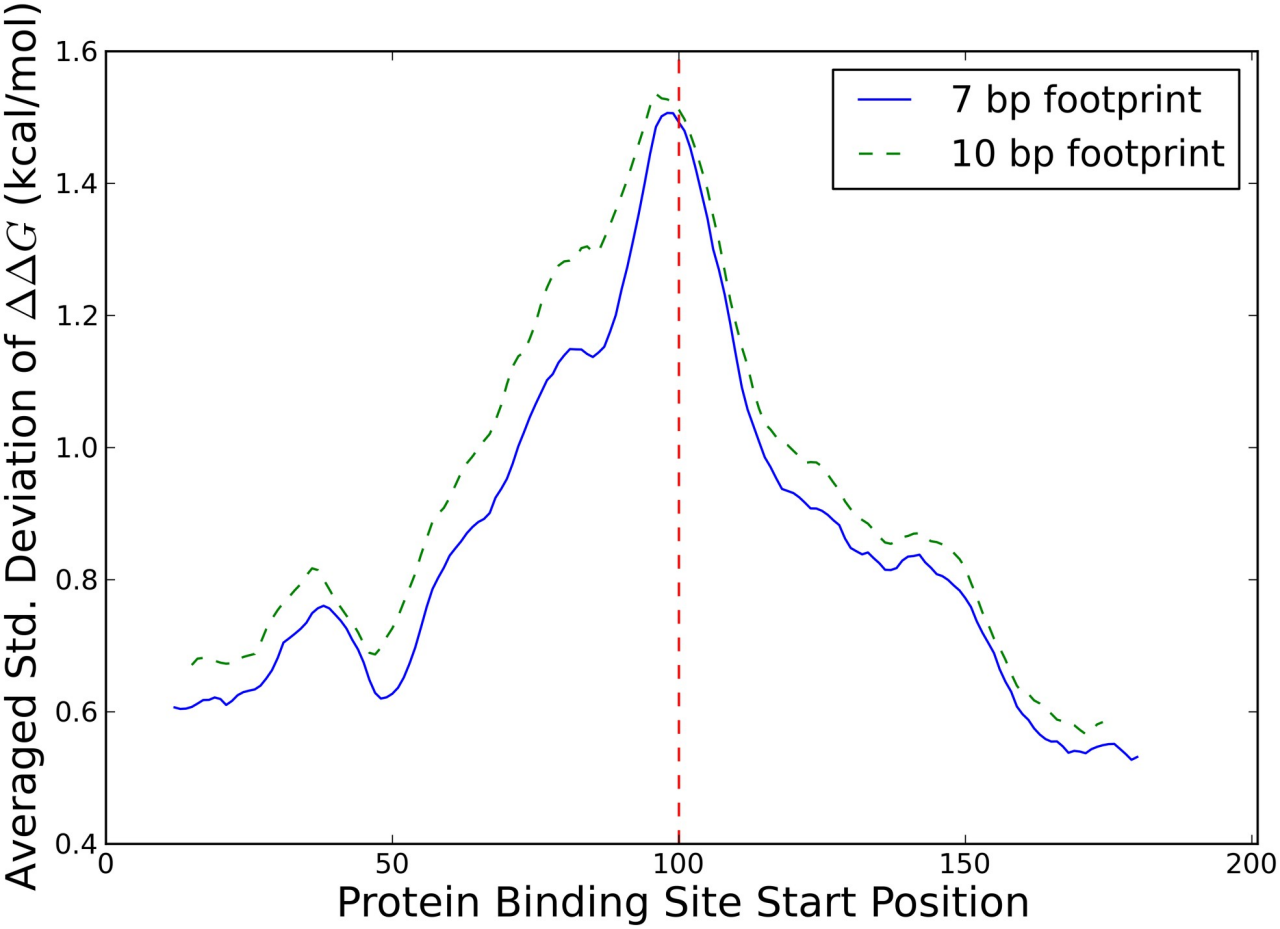
Effect of protein footprint on standard deviation of ΔΔ*G* in 201 nucleotide sequences. Standard deviations of the change in effective RNA-protein binding free energy, ΔΔ*G*, from Fig 1B (solid blue line) and Fig 1D (dashed green line) above, averaged both over the six different single nucleotide sequence alterations and a 10 base pair running average to smooth the curves. Sequence alteration location (indicated by dashed vertical red line) is static while protein binding site start position is variable.

As a first step in the investigation into secondary structure mediated effects of SNPs on RNA-protein binding, we wanted to find out if effects of sequence alterations outside the binding site on RNA-protein binding are generically possible and if so, at which distances between the sequence alteration and the protein binding site. To this end, we selected random sequences and computationally quantified the effect of a sequence alteration at the central nucleotide on the affinity of the randomly chosen RNA molecule to a hypothetical protein binding at variable locations along the molecule. We selected random sequences of lengths 101, 201, and 401 nucleotides, and saw no significant differences between the lengths. Specifically, we used the Vienna package to calculate ΔΔ*G*, or the difference in the effective free energies of binding for a protein binding to the altered and unaltered sequences taking into account the entire ensemble of RNA secondary structures (see Methods). A positive ΔΔ*G* means the alteration makes it easier for a protein to bind, while a negative ΔΔ*G* means it is harder for a protein to bind. We find that, in an ensemble of random sequences, the average of ΔΔ*G* is near zero unless a sequence alteration is directly inside a protein binding motif (see Fig 1(A) and 1(C) for 201 nucleotide data and S1(A), S1(C), S2(A) and S2(C) Figs for 101 nucleotide and 401 nucleotide data, respectively). This means that the average effect of a sequence alteration on RNA-protein binding is low, unless the sequence alteration directly impacts the protein binding motif. However, an average of zero does not necessarily imply that the effect of each individual sequence alteration is low, just that the effect of sequence alterations is symmetric. We thus next calculated the standard deviation of ΔΔ*G* and found it to be on the order of a kcal/mol even when the sequence alteration is 30-50 bp away from the protein binding site (see Fig 1(B) and 1(D) for 201 nucleotide data and S1(B), S1(D), S2(B) and S2(D) Figs for 101 nucleotide and 401 nucleotide data, respectively). This indicates that individual sequence alterations have the potential for biologically relevant effects of several kcal/mol on the binding of a protein, simply through changes in secondary structure, but that this effect is mostly symmetric for random sequences. Directly comparing standard deviations of ΔΔ*G* for the two different footprint sizes, averaging over all possible sequence changes and over 10 base pair sliding windows to reduce the noise, we also see that proteins with larger footprints are affected at slightly farther distances from an associated sequence alteration (see Fig 2 for 201 nucleotide data and S3 and S4 Figs for 101 nucleotide and 401 nucleotide data, respectively).

### Known SNPs in the human genome affect HuR binding

Given our finding that single nucleotide sequence alterations can have an effect on proteins binding to RNA through changes in secondary structure, we wished to investigate this effect in an actual genome using known SNPs. Using *in vivo* PAR-CLIP HuR binding data from three different studies [30–32], we were able to locate documented SNPs near HuR binding sites in transcript coordinates (see Methods). By utilizing a previously modified version of the Vienna Package [33] we are able to quantitatively predict the effective dissociation constant *K*_*D*_ for a single stranded RNA binding protein such as HuR as a function of RNA sequence fully taking the effects of RNA secondary structure into account. By folding genomic sequences we were thus able to determine the change in dissociation constant *K*_*D*_ for HuR binding to an RNA transcript, associated with changing the SNP from its reference to its alternate allele. We emphasize that the approach we use does not attempt to determine the dissociation constants of the protein RNA interaction from protein structure as was done in [34], but rather uses complete sets of experimentally determined protein dissociation constants [35] for unstructured RNA to predict the effective dissociation constant in the presence of RNA secondary structures. While this is still computationally challenging, it allows a transcriptome wide analysis, which more first principle based approaches would not.

**Fig 3:**
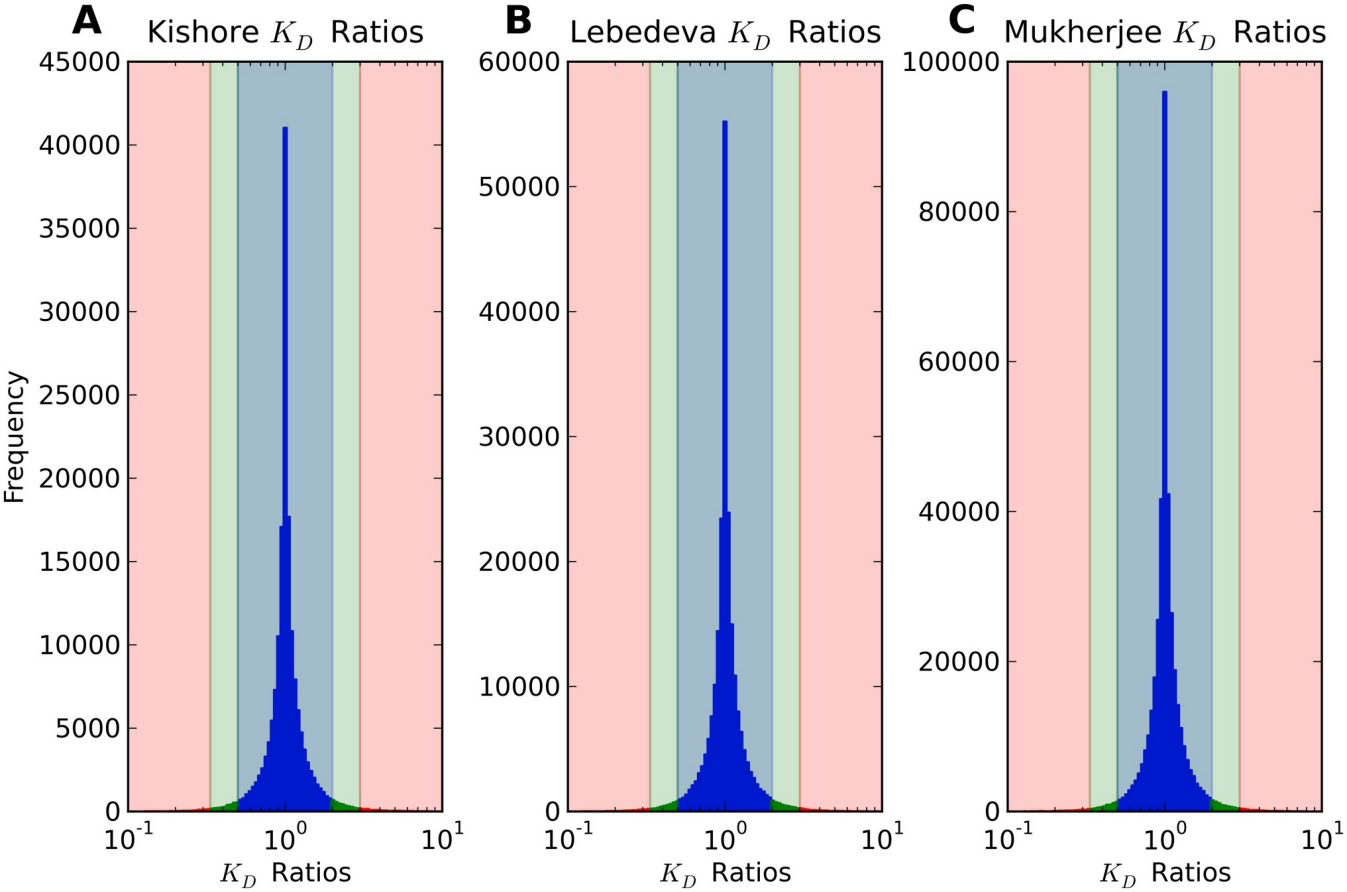
*K*_*D*_ ratios of HuR binding to 201 nucleotide sequences with and without SNPs. Histograms for the affinity ratios using (A) Kishore [30] HuR binding sites, (B) Lebedeva [31] HuR binding sites, and (C) Mukherjee [32] HuR binding sites. Affinity ratios are defined to be the dissociation constant *K*_*D*_ for HuR binding to the alternate allele of the SNP over the dissociation constant *K*_*D*_ for HuR binding to the reference sequence. Ratios larger than threefold are shown in red, ratios between two- and threefold are shown in green, and ratios less than twofold are shown in blue.

We again folded sequences of length 101, 201, and 401 nucleotides. Taking the ratio of *K*_*D*_’s for HuR binding to an RNA transcript with the reference and the alternate allele of the SNP allows us to measure the effect of SNPs on HuR binding to RNA. Histograms of these affinity ratios for both data sets are shown in Fig 3. We find that while a majority of ratios are close to one, for some HuR binding sites near known SNPs in the tail of the distribution the predicted affinity of HuR to the transcript changes by tenfold or higher (see the minimal and maximal affinity ratios in Table 1) depending on the allele of the SNP. While this tail of highly impactful SNPs is a small fraction of the whole, it is still on the order of tens of thousands of SNPs.

**Table 1:** Effects of SNPs on HuR binding affinity in 201 nucleotide sequences.

**Data Set**	**Ratio Count >1**	**Ratio Count <1**	**Ratio Min**	**Ratio Max**
Kishore	88443	82921	0.0268	162.6
Lebedeva	119408	114117	0.0384	44.60
Mukherjee	208486	198778	0.0375	44.60
	**Binomial p-value**	**Ratios >1 Mean**	**Ratios <1 Mean**	**All Ratios Mean**
Kishore	6.944 ⋅ 10^−41^	1.277	0.8551	1.072
Lebedeva	3.423 ⋅ 10^−28^	1.267	0.8530	1.064
Mukherjee	1.487 ⋅ 10^−52^	1.259	0.8572	1.062

Data features for *K*_*D*_ ratios of SNPs near HuR binding sites from the Kishore [30], Lebedeva [31], and Mukherjee [32] HuR PAR-CLIP data sets.

**Fig 4:**
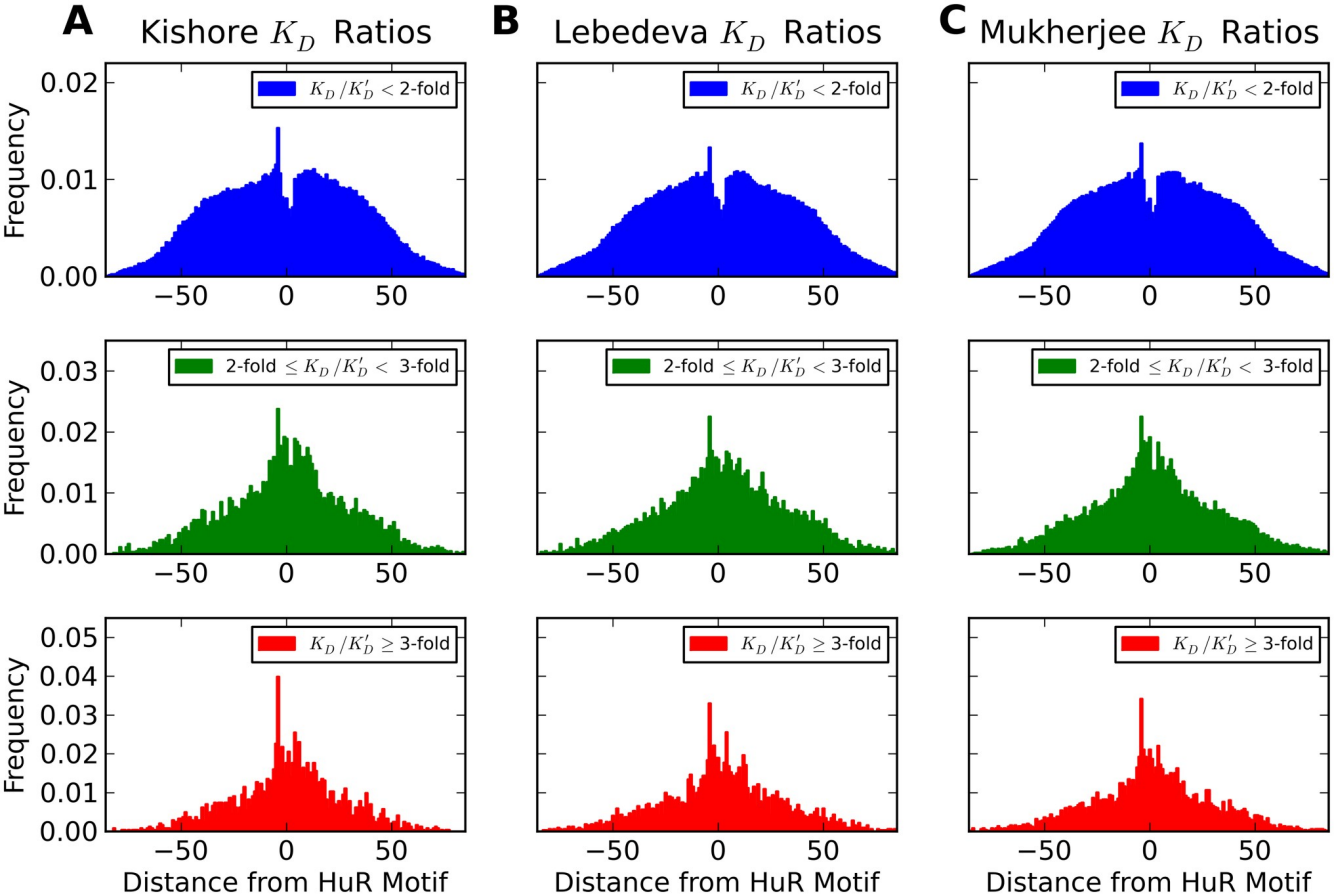
Effect of distance from motif on change of binding affinity due to SNPs in 201 nucleotide sequences. Histograms of distances of SNP locations from the center of the nearest HuR binding motif for (A) the Kishore data set, (B) the Lebedeva data set, and (C) Mukherjee data set for different strengths of their effects on HuR binding. Distances where the SNP is upstream of the motif are negative. The top (blue) histograms are of SNPs with an absolute fold change (positive or negative) in binding affinity less than 2, the middle (green) histograms are of SNPs with a fold change between 2 and 3, and the bottom (red) histograms are of SNPs with a fold change of 3 or greater.

Next, we wanted to know how distance between the SNP and the HuR binding site affects the binding affinity ratio associated with the SNP. Fig 4 shows histograms of SNP positions relative to the nearest HuR binding site for different ranges of *K*_*D*_ ratios. The most obvious observation from these histograms is that in all data sets SNPs occur less frequently than expected on the HuR binding motifs themselves, with the first nucleotide in the motif occurring slightly more often than the others, and the first nucleotide upstream of the motif enriched in SNPs. This is unsurprising, since we would expect such an important binding motif to be evolutionarily conserved. In addition to this general trend, we find that although the distributions for SNPs with different affinity fold-changes become narrower with higher strength (standard deviations for the blue, green, and red curves of Fig 4(A) being 33.25±.06, 28.23±.26, and 25.28±.36, of Fig 4(B) being 34.43±.05, 29.76±.23, and 27.94±.35, and of Fig 4(C) being 34.19±.04, 29.50±.18, and 27.89±.28, respectively) even for the highly impactful SNPs (with a fold change of three-fold or larger) a majority fall outside of the HuR binding motifs. This further supports the idea that a SNP does not need to be directly on a protein binding motif to impact RNA-protein binding, and can affect RNA-protein binding at a distance through changes in secondary structure.

### The effect of SNPs on protein binding appears to be under selection *in vivo*

**Fig 5:**
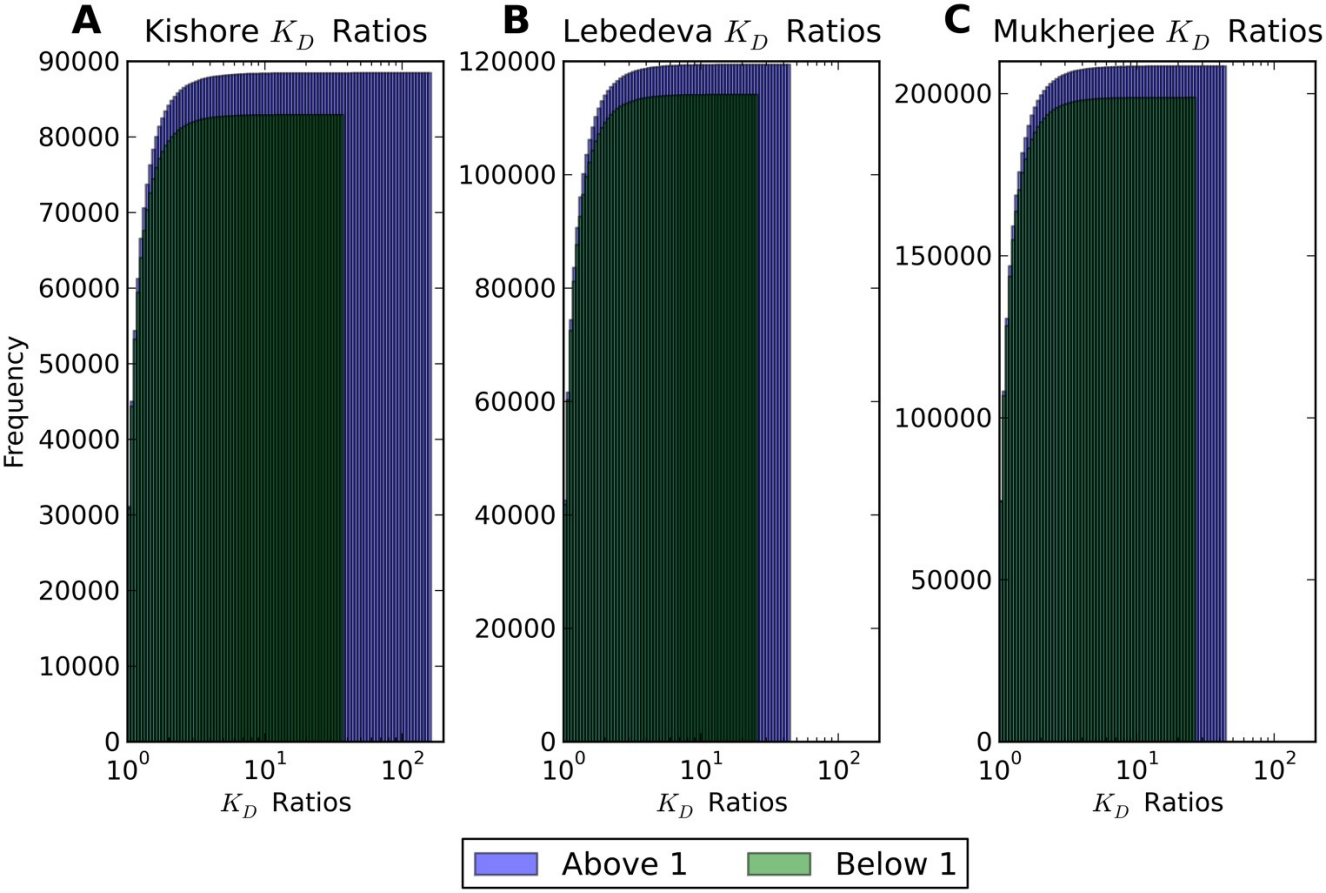
Cumulative histogram of *K*_*D*_ ratios in 201 nucleotide sequences. Cumulative histograms for the affinity ratios using (A) Kishore [30] HuR binding sites, (B) Lebedeva [31] HuR binding sites, and (C) Mukherjee [32] HuR binding sites. Ratios less than 1 are reciprocated to be larger than 1, and shown in dark (green), while ratios naturally larger than 1 are shown in lighter color (blue).

In the previous two sections we demonstrated that SNPs have the ability to affect the interactions between RNA and regulatory proteins. This leaves the question of whether or not the effect of SNP alleles on RNA protein interactions has any functional relevance *in vivo*. In order to address this question, we asked if there is any evidence for selection for or against protein binding. We reasoned that if the effects of sequence on protein RNA interactions do not play a functional role, protein RNA affinity should increase as often as decrease when changing the sequence from the reference to the alternate allele of a SNP. In contrast to this expectation under the null assumption of no functional relevance, we in fact find a significant asymmetry in the direction of SNPs’ effect on protein binding, which we thus take as an indication of functional relevance. It is clear from the cumulative distributions in Fig 5 (where ratios below 1 are reciprocated) that SNPs with affinity ratios above 1 are more prevalent, and that ratios above 1 have a larger maximum effect for sequences of this length. Since we take affinity ratios to be the *K*_*D*_ for binding with the alternate allele over the *K*_*D*_ for binding with the reference allele, this indicates that changing the SNP from its reference to its alternate allele is more likely to make it harder for a protein to bind. This effect is quantified in Table 1, which notes that if we assume a binomial distribution for SNPs with affinity ratios above and below 1, we can reject the null hypothesis of a 50/50 split with p-values of 6.9 ⋅ 10^−41^, 3.4 ⋅ 10^−28^, and 1.5 ⋅ 10^−52^ for the Kishore, Lebedeva, and Mukherjee data set, respectively.

## Discussion

SNPs have long been associated with disease, but the role of non-coding and synonymous SNPs in altering phenotypes is still unclear. Using a modified version of the Vienna Package we have verified that SNPs can affect RNA protein binding affinity by modifying RNA structures from tens of nucleotides away, and performed a genome wide analysis of the effect of SNPs on the binding affinity of the RNA binding protein HuR. Our analysis shows that SNPs can affect the affinity of a protein binding to RNA by many fold and that a significant number of HuR binding sites in the human genome, mostly in UTRs and introns, are strongly affected in their binding affinity by nearby SNPs. We also identify an asymmetry in the effect of SNPs on HuR binding, implying that the effect of SNPs on RNA structure might be under selective pressure in the human genome, at least in the case of HuR binding sites.

SNPs have been known to affect the structure of RNAs, in particular many RiboSNitches, or SNPs with a large impact on RNA secondary structure, have been identified [23], but characterizing the effect of these SNPs and revealing the ways in which they cause disease remains a challenge. Several studies and web servers use various minimum free energy (MFE) or partition function distance measures to characterize which SNPs have a high impact on global or local RNA structure [36–38], but our analysis is to our knowledge the first to identify the genome-wide effects of the structural change caused by a SNP on a given RNA binding protein and give the change in binding affinity associated with the different alleles of a SNP. To encourage individual experimental validation of our findings, the full data for the Lebedeva data set, including sequences of length 201 and calculated binding affinities, is provided (see S1 Table).

Interference with RNA protein binding is a clear link between SNPs and causes of disease. The disruptive nature of SNPs on the human genome is evident both from SNPs occurring less frequently on HuR binding sites and from the asymmetry in the effect of SNPs on HuR binding affinities, which suggests that a SNP is more likely to disrupt protein binding than enhance it. This trend is similar to a trend observed in previous studies of the effect of SNPs on the MFE of RNA secondary structures, which found that the effects of SNPs skew RNAs towards higher free energy structures [39]. This trend suggests that existing RNA secondary structure is optimized to leave important single stranded RNA binding protein motifs unpaired, and SNPs have the ability to disrupt these naturally optimized configurations.

While we interpret this asymmetry to be the result of evolutionary selection preferring uninhibited HuR binding sites, biased HuR binding data could provide another possible explanation for the observed asymmetry. If an experimental binding site is higher affinity in the presence of the reference allele, it has a higher chance of appearing in PAR-CLIP data, and it will appear as impeded in the presence of the SNP. In the opposite case, if a binding site is lower affinity in the presence of the reference allele, it is less likely to appear in the PAR-CLIP data, which could contribute to the asymmetry. To disprove this alternate explanation, an analysis of only the heterozygous and homozygous alternate SNPs in HEK 293 cells (which should be immune to this selection bias or be biased in the opposite direction, respectively) was performed, but the number of these SNPs near HuR sites was not high enough to draw statistically significant conclusions. We also find that with increasing length of the sequence fragment studied there is a reduction in effect asymmetry and the number of highly impactful SNPs (see S2 Table), which we attribute to non-specific binding. Indeed, when we perform a hard constraint analysis (as we did on random sequences above) which only measures the effect of a SNP on the central binding site, we find no reduction of asymmetry or number of impactful SNPs with increased sequence length.

We have shown that individual SNPs can disrupt specific RNA protein binding sites, but many SNPs in a genome could all contribute to lower RNA protein binding efficiency and cause disease on a genome-wide or many gene scale. SNPs are typically not independent of each other but appear together in haplotypes. The combined effect of SNPs in a haplotype is not considered in this analysis, but it is reasonable to expect in general that sequence changes at multiple positions lead to even larger structural changes and thus stronger effects on protein binding. While this is the general expectation, it is also possible that multiple SNPs could have compensatory effects, but a systematic search for compensatory effects on protein binding is beyond the scope of this work. It is also clear from our analysis that SNPs need not be directly on a protein binding motif, or even within 50 nucleotides of a motif, to disrupt binding. This wide range of effect suggests that future studies on the structural effect of SNPs examine SNPs in a wide radius of their target feature.

While we have focused here on changes of individual nucleotides, other genomic variations, i.e., short insertions or deletions, might have even stronger effects on protein affinity of mRNAs and will be the subject of future investigations. Post-transcriptional modifications to mRNAs could also cause structural changes analogously to an allele change in a SNP, and once energy parameters for post-transcriptional modifications are available the analysis performed here for SNPs could be applied to them as well. Similarly, it will be interesting to investigate if similar effects apply to proteins with preferences for double stranded RNAs.

## Materials and methods

### RNA secondary structure prediction and RNA-protein binding

Although RNA is synthesized as a single stranded molecule, its constituent bases can pair with each other, ultimately leading to formation of complicated 3D structures. To perform our analysis of the effect of SNPs on RNA-protein binding we must model these structures *in silico*. In principle, a complete 3D model, or tertiary structure, is required to fully describe an RNA. However, many properties of RNA structure can already be understood at the level of secondary structure, i.e., the list of base pairs in the molecule [40]. The secondary structure is modeled by the Vienna Package [41], state-of-the-art software which takes into account base pairing and nearest-neighbor stacking energies when modeling secondary structure. We take a similarly simplified approach to RNA-protein binding, modelling a bound protein by simply forcing any bound bases to remain unpaired and adding a protein binding energy for those bound configurations [42]. The Vienna RNA Package uses recursion relations to efficiently fold RNAs in *O*(*N*^3^) time for RNAs of length *N* and allows the exclusion of individual bases from the folding through its constraint folding capabilities. We also make use of a previously published altered version of the Vienna Package that takes single stranded RNA binding proteins and their experimentally determined sequence-dependent binding energies [35] into account in the recursion relations themselves, and calculates the dissociation constant *K*_*D*_ of a known protein to an RNA of a given sequence fully taking into account RNA secondary structures [33, 43]. We note that while the quality of computational secondary structure prediction via determination of the minimum free energy structure can be questionable, all our calculations evaluate partition functions over the entire Boltzmann ensemble of all RNA secondary structures, which are much more reliable [44]. Also, it is important to note that while we do not explicitly allow non-canonical base pairs in our secondary structure predictions, their effect on the secondary structure is at least partially taken into account by the Vienna package in the measured free energy parameters for short interior loops exhibiting such base pairs.

### Genomic data sets and tools

To investigate the role of SNPs on protein binding we used all human (GRCh38.p7) SNPs from dbSNP (build 151) downloaded in VCF format [45]. SNPs were formatted using vcftools [46] and transcript coordinates were obtained using Variant Effect Predictor (VEP) [47]. We analyzed the effect of SNPs on three different HuR PAR-CLIP data sets, one from Kishore *et al*. [30], one from Lebedeva *et al*. [31], and one from Mukherjee *et al*. [32], which were all downloaded from the doRiNA database [48]. HuR binding sites were matched to transcripts using the ensembldb bioconductor package [49]. HuR binding affinities to different 7-mers were obtained from RNAcompete data [35]. All reference transcript sequences were obtained from Ensembl BioMart [50].

### Quantification of the effect of sequence changes on protein binding

**Fig 6:**
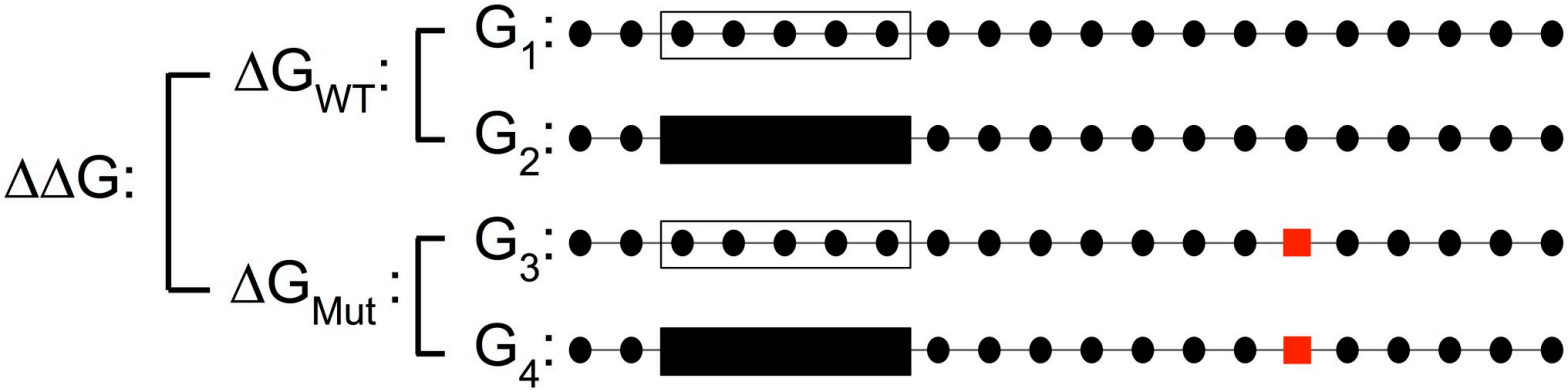
Possible configurations of a SNP and protein on RNA. The four different configurations of an RNA subject to sequence variation interacting with a protein: wild type sequence not bound by a protein, wild type sequence bound by a protein, mutant sequence not bound by a protein, or mutant sequence bound by a protein. Lines represent RNA backbones, and black dots represent bases. Transparent and opaque boxes represent unbound and bound protein binding sites, and red squares represent a change in nucleotide identity between the wild type and mutant sequences. Bases bound by a protein cannot base pair, but the base that differs between wild type and mutant can.

To investigate the effect of single nucleotide sequence changes on proteins binding in the presence of RNA secondary structure we computed the changes in ensemble Gibbs free energy for proteins binding to random sequences at different positions using the Vienna Package. We selected 100 random sequences each for varying lengths (101, 201, and 401 nucleotides) with equal probabilities for all four nucleotides. Results for sequences of length 201 nucleotides are shown in the text while results for sequences of length 101 and 401 are shown in the supplementary material (see S1, S2, S3 and S4 Figs). Results for the 201 nucleotide sequence length were also replicated using RNAstructure, and we found very close agreement between the two software packages (see S5 and S6 Figs) [51]. For each of these “wild type” sequences we considered “mutated” sequences that differ from wild type only in the identity of the central nucleotide. Then we used the constrained folding feature of the Vienna Package as described above to calculate free energies for four different configurations: the wild type sequence without a protein, the wild type sequence with a protein bound, the mutant sequence without a protein, and the mutant sequence with a protein bound. We used protein footprints of 7 nucleotides (the same as HuR) and 10 nucleotides to interrogate how the effect depends on footprint size. We then calculated the difference in free energy to bind a protein, Δ*G*_WT_ and Δ*G*_Mut_ for the wild type and mutant sequence, respectively (see Fig 6). This difference between the free energy of the unconstrained ensemble of all RNA secondary structures and the ensemble of all RNA secondary structures in which the binding site of the protein remains unpaired can also be interpreted as Δ*G*_WT_ = −*k*_*B*_ *T* log(*p*_WT_), where *p*_WT_ is the probability that the entire binding site of the protein is unpaired. As a quantitative measure of the effect of the sequence alteration on protein binding, we then calculated the difference ΔΔ*G* = Δ*G*_Mut_—Δ*G*_WT_. For each sequence we computed this quantity for each of the six possible combinations of wild type and mutant nucleotide at the central position and for every possible position of the protein binding site along the molecule. Finally, we calculated the average and standard deviation of ΔΔG over the 100 random sequences considered.

### Identifying SNPs near HuR binding sites

We identified SNPs near HuR binding sites by first matching the genomic coordinates of SNPs to transcript coordinates. Given a list of SNPs in VCF format, VEP provides each SNP’s associated Ensemble transcript ID (ENST) and cDNA position (if the SNP is transcribed), as well as its allele. The resulting list of SNPs with transcript coordinates was further filtered for only transcripts annotated by Ensembl as canonical, which are generally chosen as the transcript with the longest coding sequence when given a choice between isoforms. HuR binding sites were also mapped to ENSTs and the list of SNP transcript coordinates and HuR transcript coordinates was compared. If a SNP was found to be within 40 bases on either side of the middle of an HuR binding site it was considered a candidate for RNA secondary structure folding analysis. From this data we were also able to examine the positional distribution of SNPs around HuR binding sites.

### Determining the effect of SNPs on binding affinity

To determine the effect of SNPs on binding affinity as mediated by RNA secondary structure we used a modified version of the Vienna Package that incorporates the effect of single stranded protein binding on RNA secondary structure calculations [33]. We first determined the 7 bp motif within the PAR-CLIP binding site (usually ∼40 bp) that HuR has the highest affinity for using RNAcompete data [35], and then folded a stretch of the RNA transcript centered on this motif for the reference sequence and the SNP-altered sequence. We fold sequences of length 101, 201, and 401 nucleotides for each motif, and results for sequences of length 201 are again shown in the main text while results for sequences of length 101 and 401 are shown in the supplementary material (see S7, S8, S9, S10, S11 and S12 Figs). Although folding longer sequences could improve the accuracy of our calculated structures, longer sequences quickly become computationally intractable. Our modified version of the Vienna Package is able to determine the dissociation constant *K*_*D*_ for HuR binding to any sequence (taking into account altered secondary structure), and by taking a ratio of these dissociation constants we are able to quantify the effect of SNPs on HuR binding due to changes in secondary structure. We take the ratio of dissociation constants to be the dissociation constant of the alternate allele over the dissociation constant of the reference allele.

## Supporting information

S1 Table The full data for the Lebedeva data set, including sequences of length 201 and binding affinities. (XLSX)

S2 Table Effects of SNPs on HuR binding affinity in 101, 201, and 401 nucleotide sequences. (XLSX)

S1 Fig Effect of SNPs in random 101 nucleotide sequences on protein binding. (TIF)

S2 Fig Effect of SNPs in random 401 nucleotide sequences on protein binding. (TIF)

S3 Fig Effect of protein footprint on standard deviation of ΔΔ*G* in 101 nucleotide sequences. (TIF)

S4 Fig Effect of protein footprint on standard deviation of ΔΔ*G* in 401 nucleotide sequences. (TIF)

S5 Fig Effect of SNPs in random 201 nucleotide sequences on protein binding calculated using RNAstructure. (TIF)

S6 Fig Effect of protein footprint on standard deviation of ΔΔ*G* in 201 nucleotide sequences calculated using RNAstructure. (TIF)

S7 Fig *K*_*D*_ ratios of HuR binding to 101 nucleotide sequences with and without SNPs. (TIF)

S8 Fig *K*_*D*_ ratios of HuR binding to 401 nucleotide sequences with and without SNPs. (TIF)

S9 Fig Effect of distance from motif on change of binding affinity due to SNPs in 101 nucleotide sequences. (TIF)

S10 Fig Effect of distance from motif on change of binding affinity due to SNPs in 401 nucleotide sequences. (TIF)

S11 Fig Cumulative histogram of *K*_*D*_ ratios in 101 nucleotide sequences. (TIF)

S12 Fig Cumulative histogram of *K*_*D*_ ratios in 401 nucleotide sequences. (TIF)
