# Supplementary figures and images for: Single nucleotide polymorphisms affect RNA-protein interactions at a distance through modulation of RNA secondary structures
Source: PLoS Comput Biol. 2020 May 7;16(5):e1007852. doi: 10.1371/journal.pcbi.1007852 (PMC7237046; doi:10.1371/journal.pcbi.1007852)

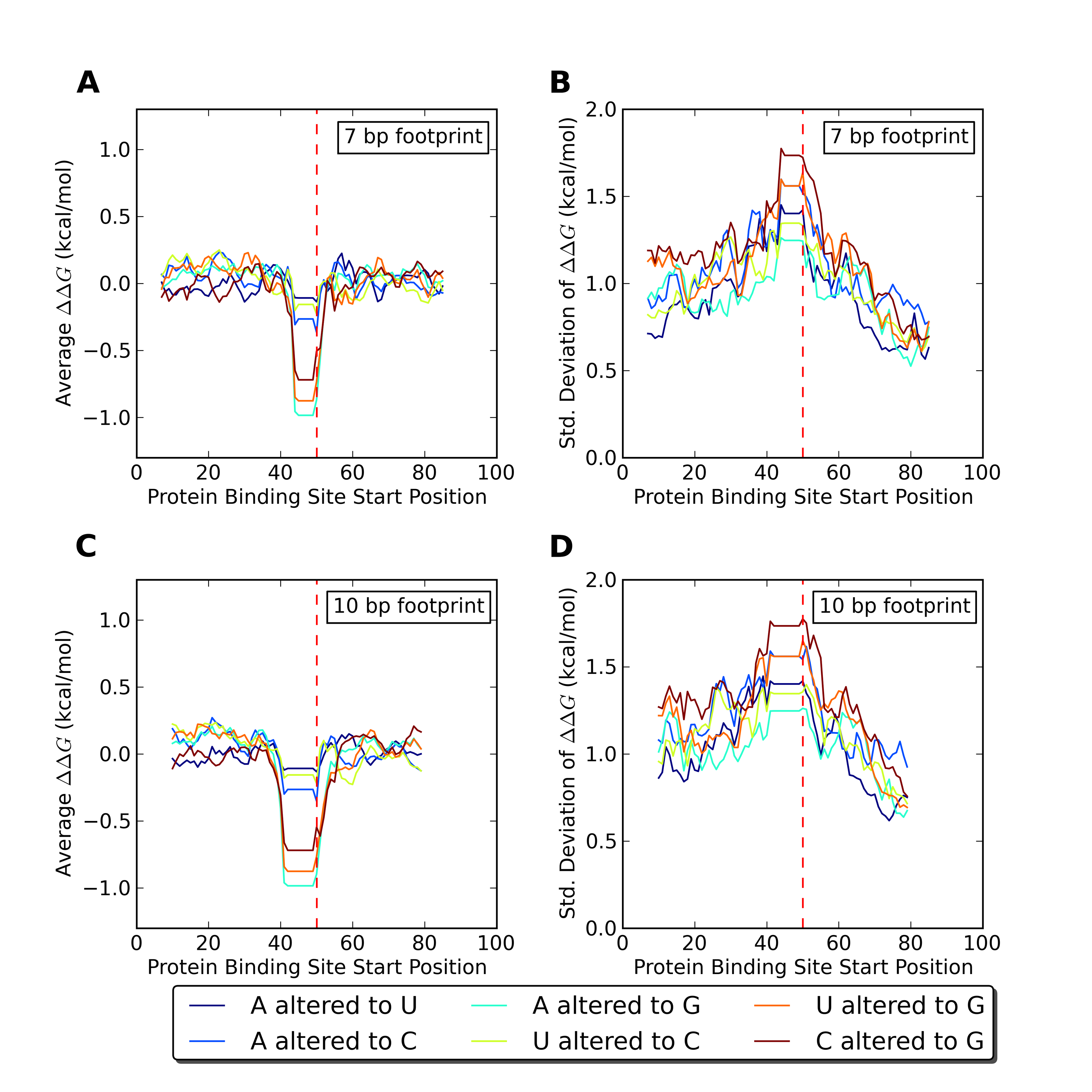

Supplement: S1 Fig — (TIF) [file pcbi.1007852.s003.tif]

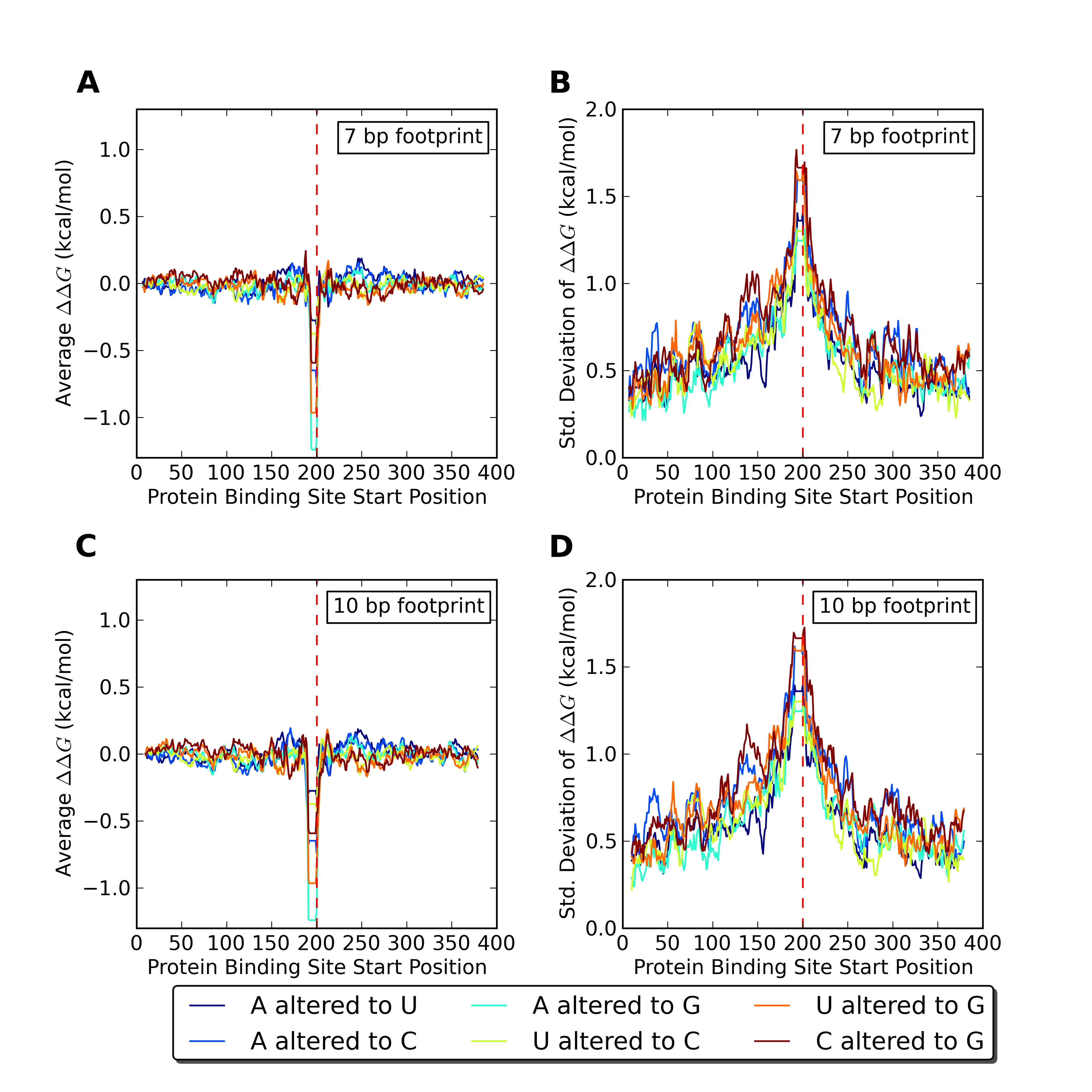

Supplement: S2 Fig — (TIF) [file pcbi.1007852.s004.tif]

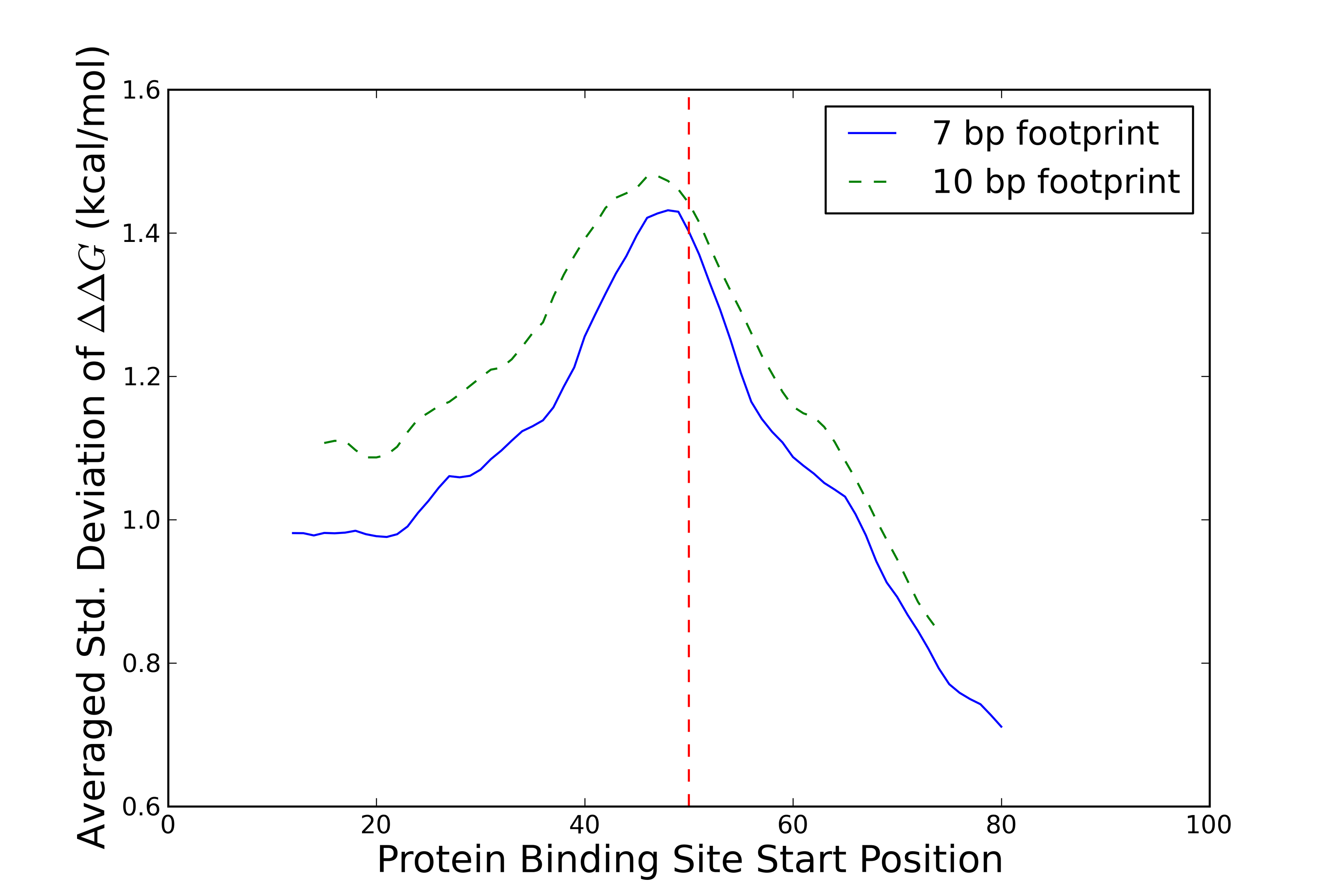

Supplement: S3 Fig — (TIF) [file pcbi.1007852.s005.tif]

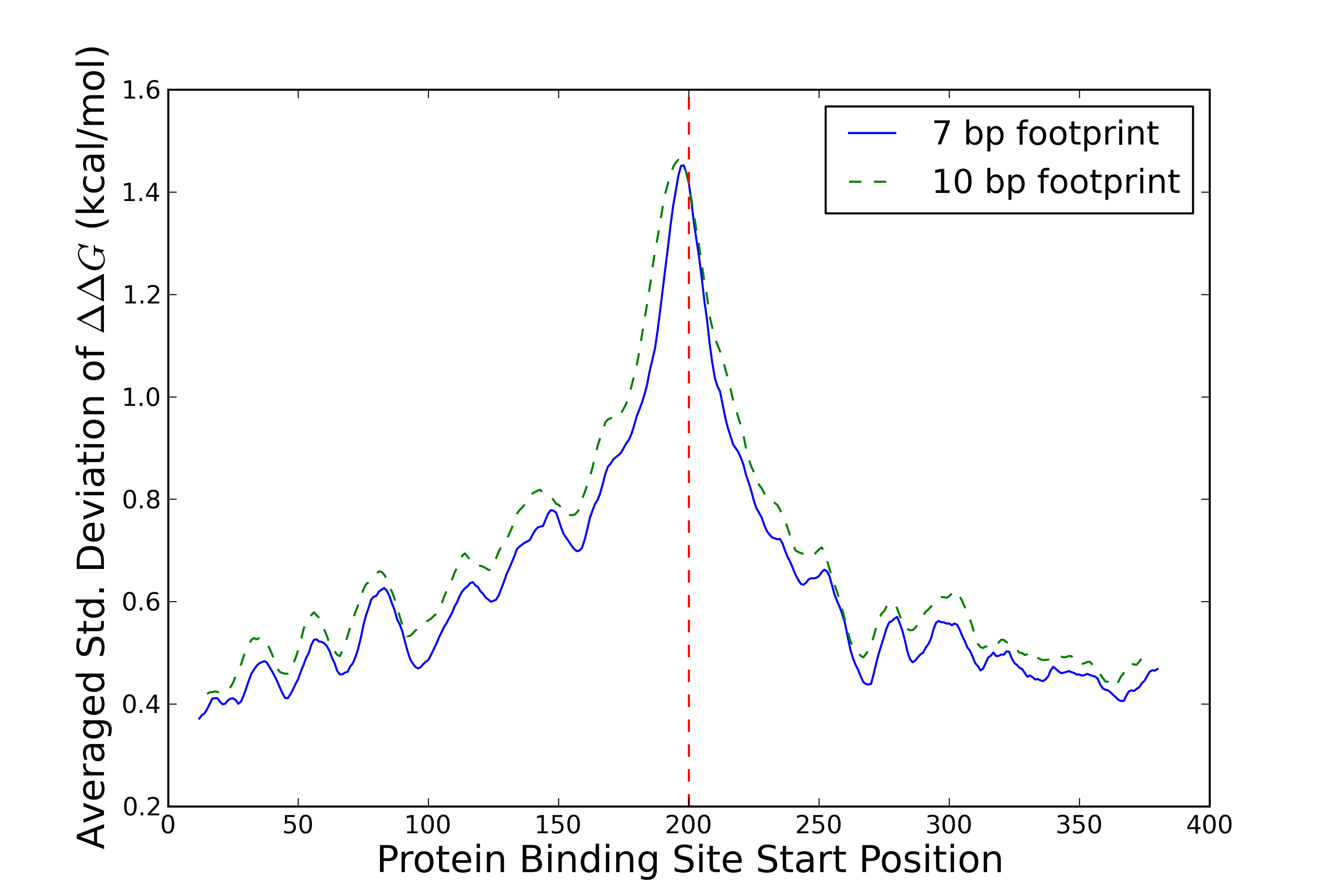

Supplement: S4 Fig — (TIF) [file pcbi.1007852.s006.tif]

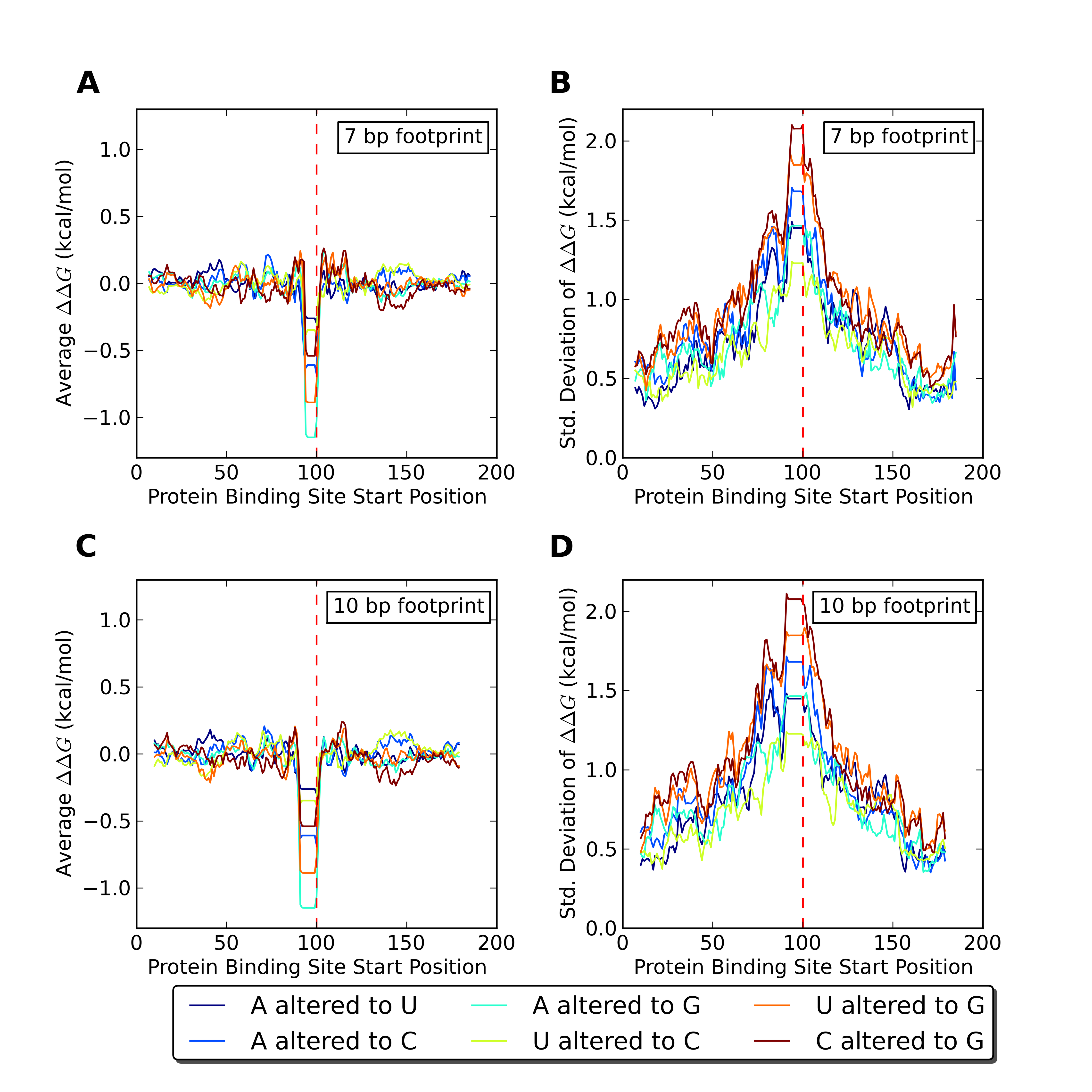

Supplement: S5 Fig — (TIF) [file pcbi.1007852.s007.tif]

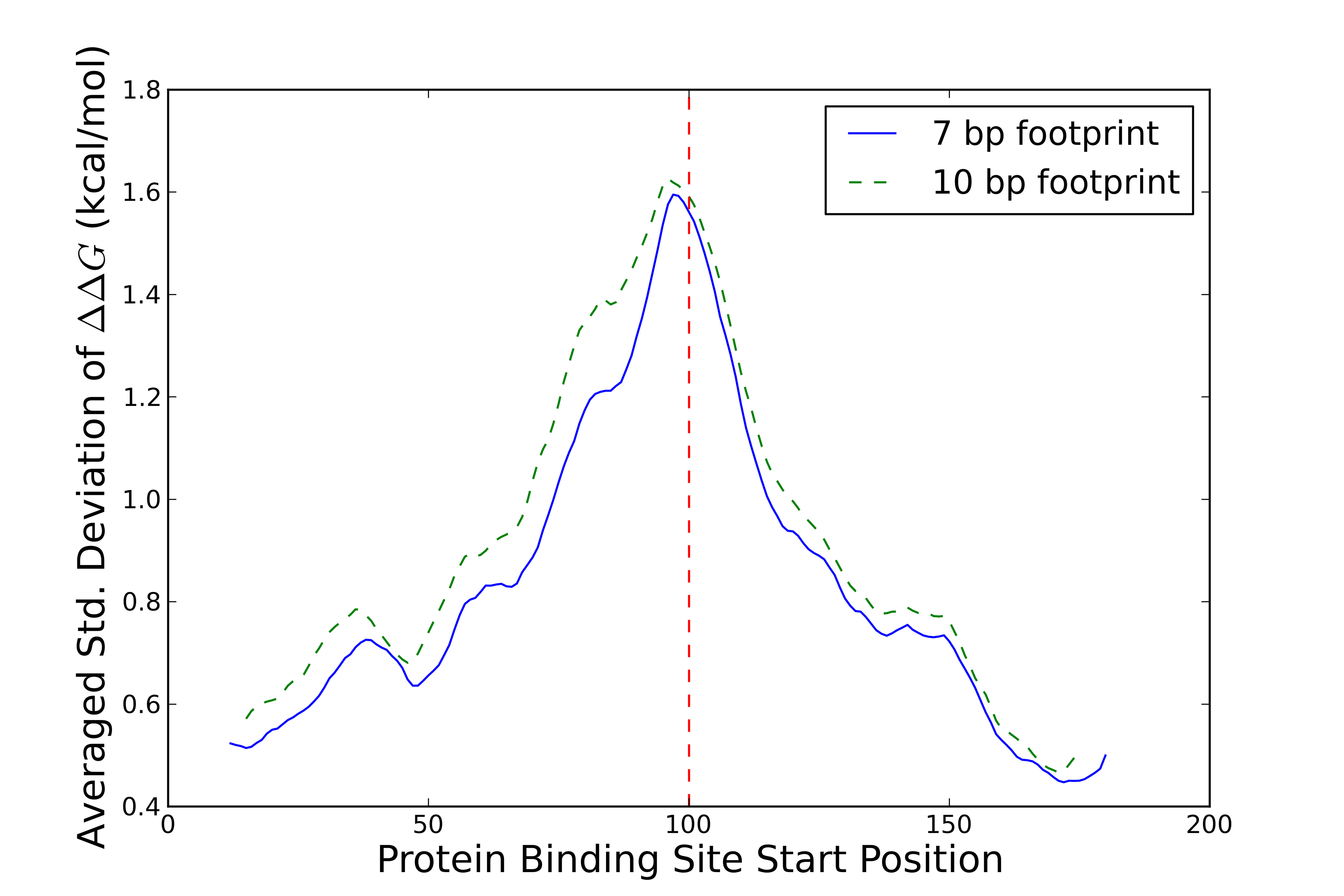

Supplement: S6 Fig — (TIF) [file pcbi.1007852.s008.tif]

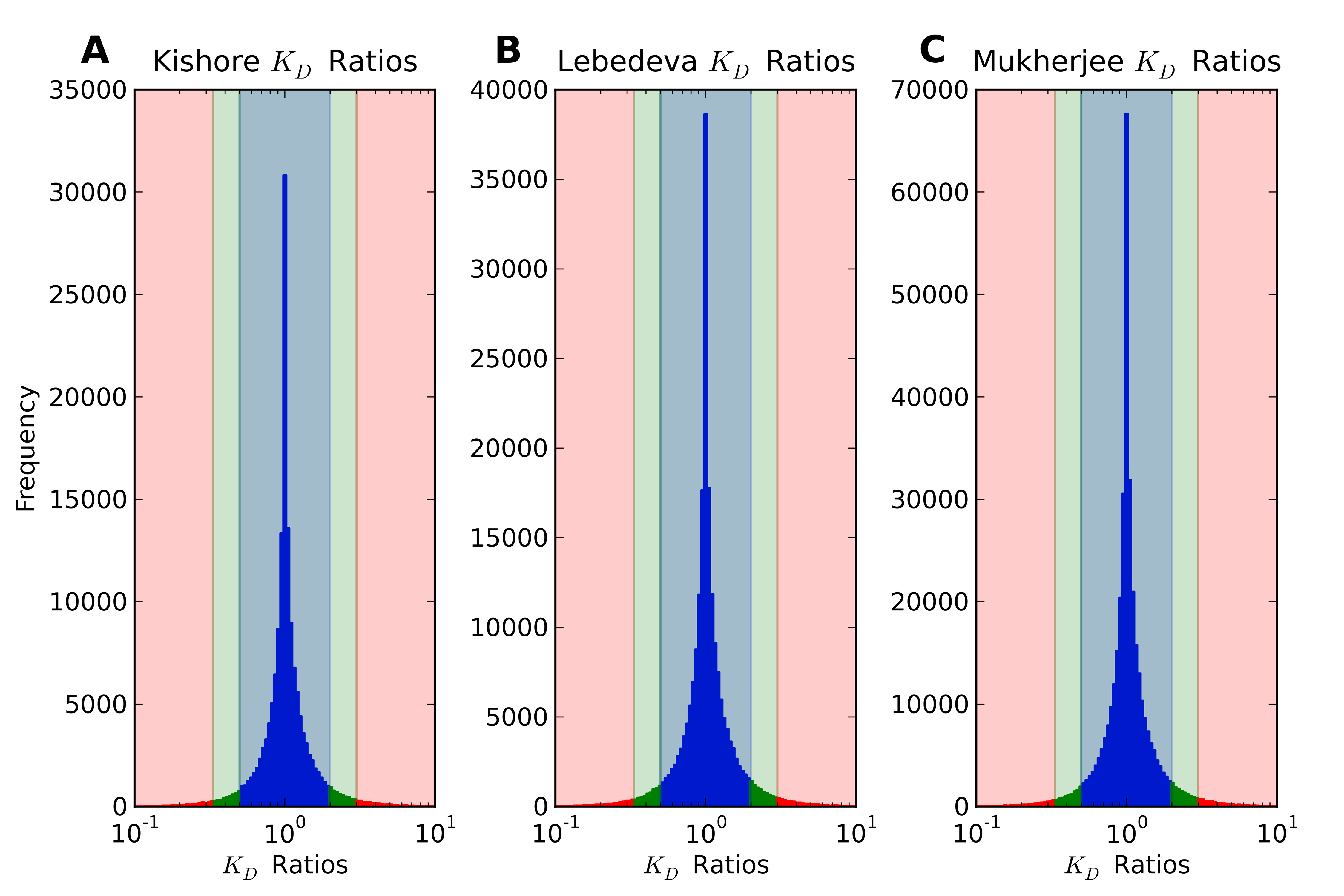

Supplement: S7 Fig — (TIF) [file pcbi.1007852.s009.tif]

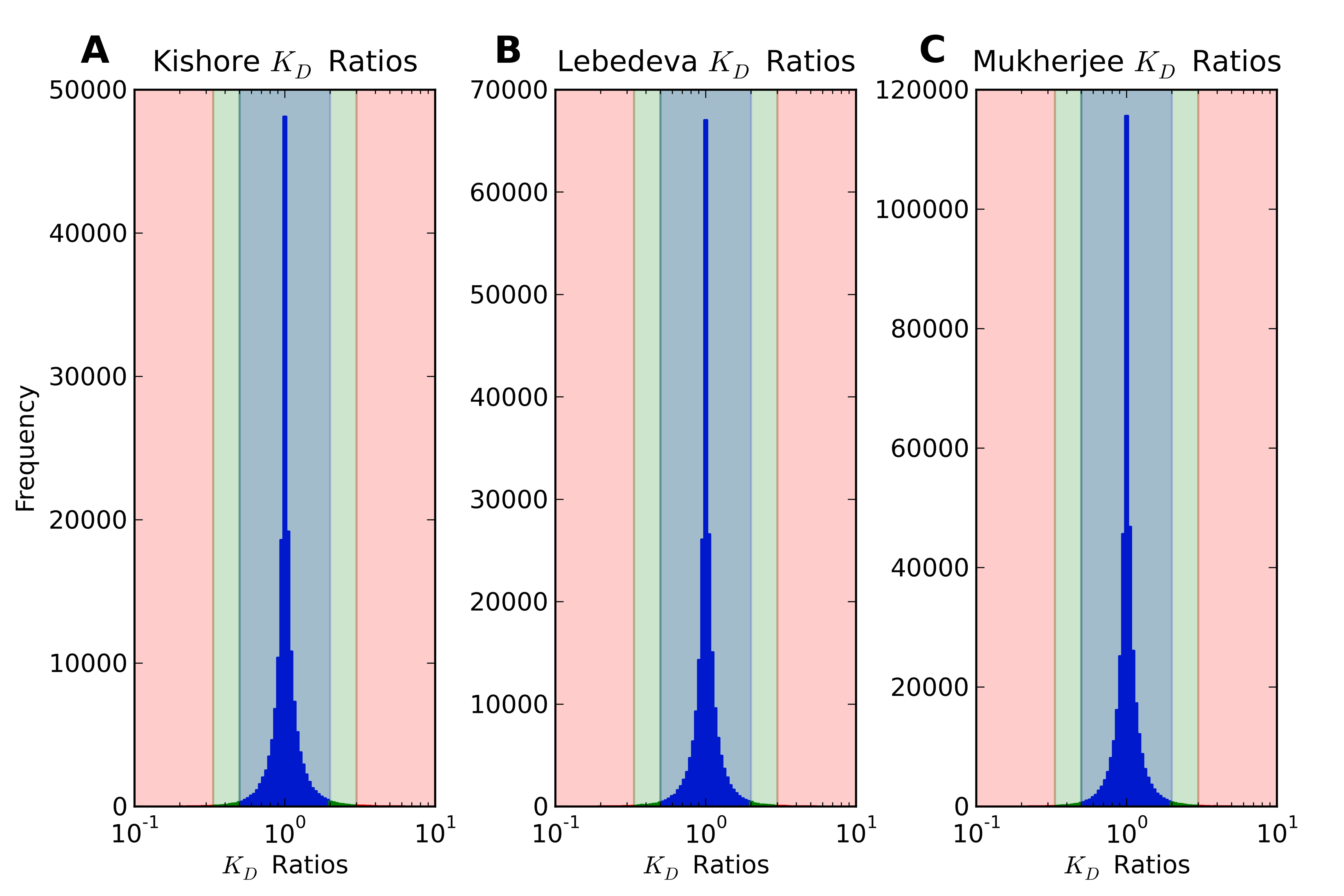

Supplement: S8 Fig — (TIF) [file pcbi.1007852.s010.tif]

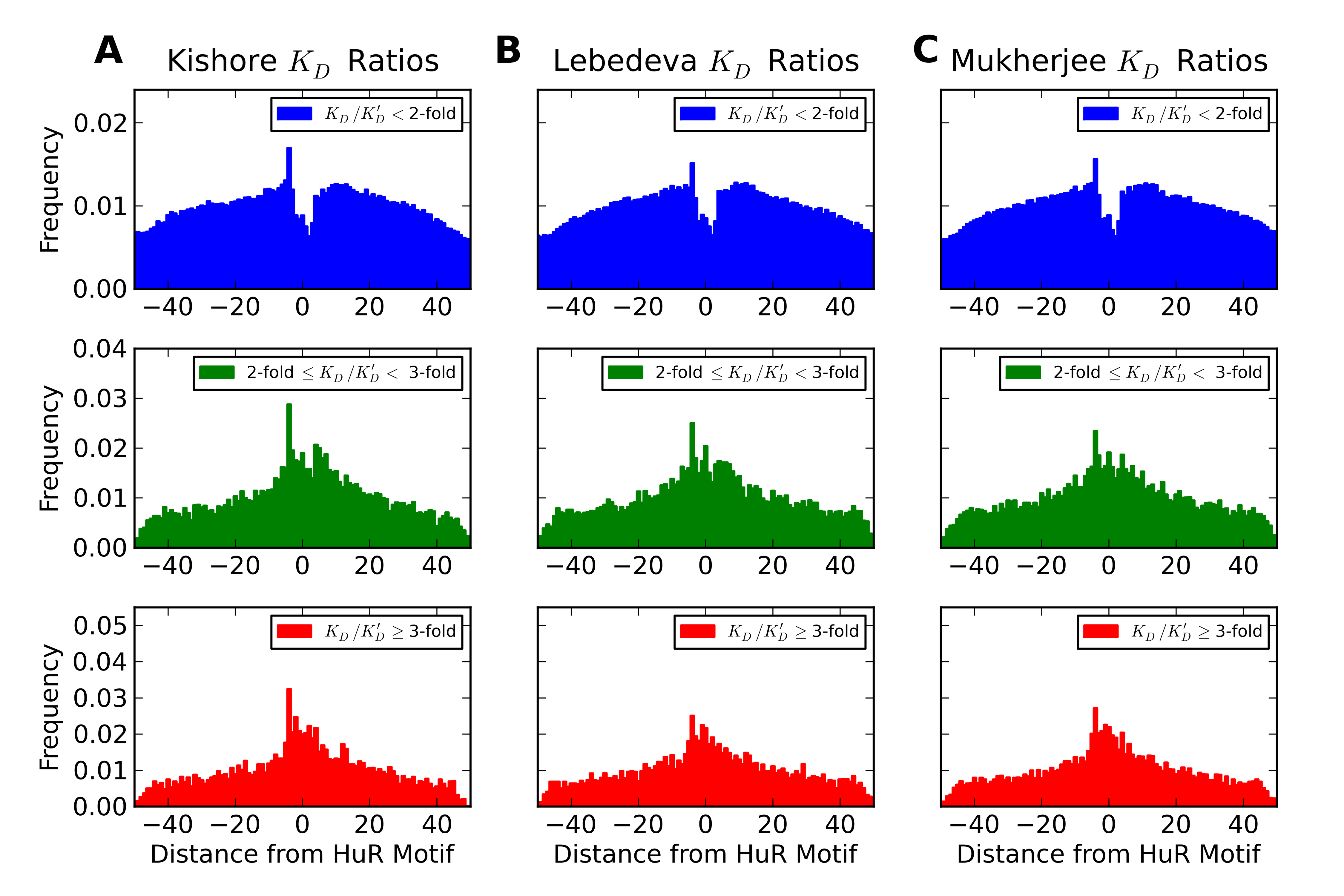

Supplement: S9 Fig — (TIF) [file pcbi.1007852.s011.tif]

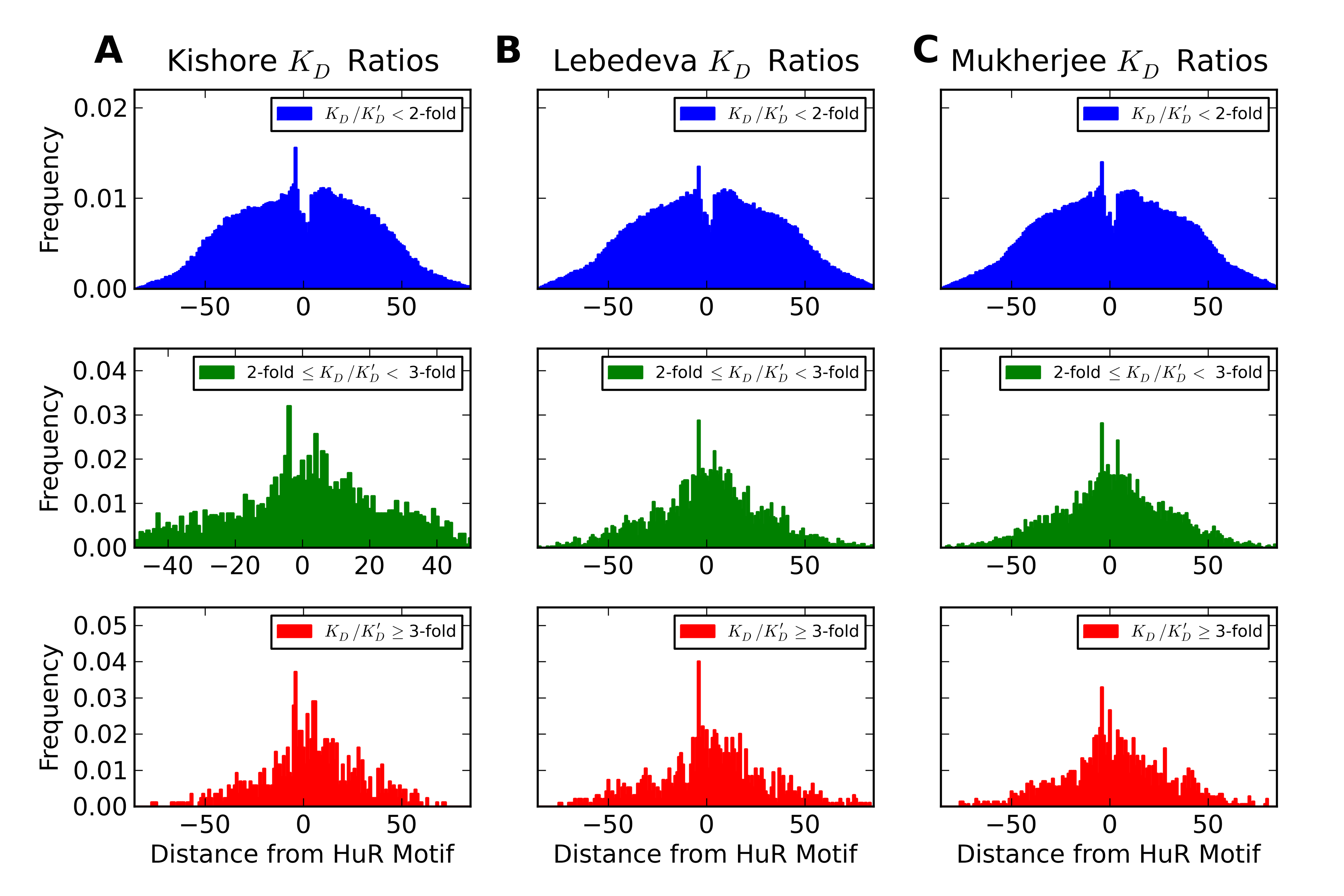

Supplement: S10 Fig — (TIF) [file pcbi.1007852.s012.tif]

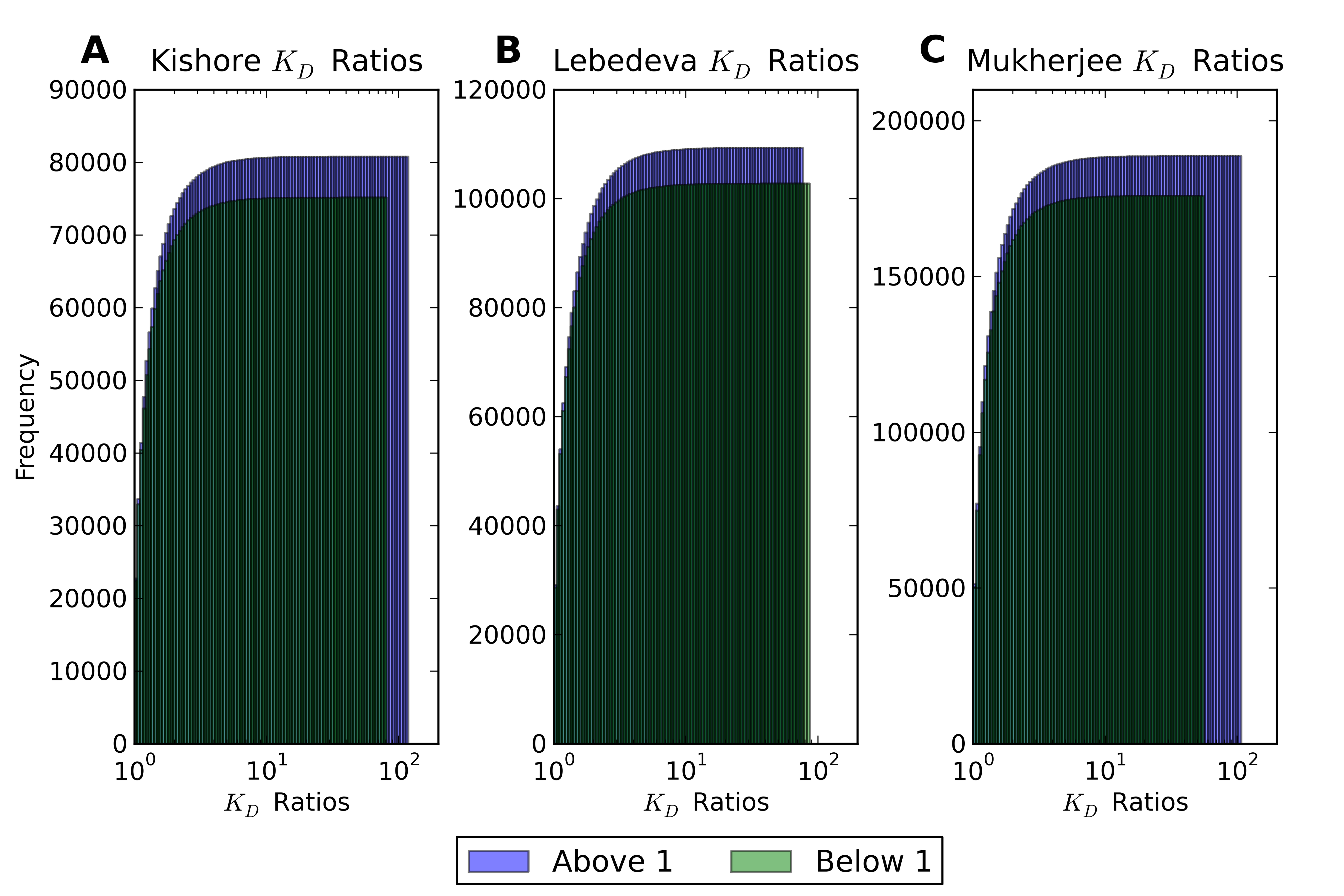

Supplement: S11 Fig — (TIF) [file pcbi.1007852.s013.tif]

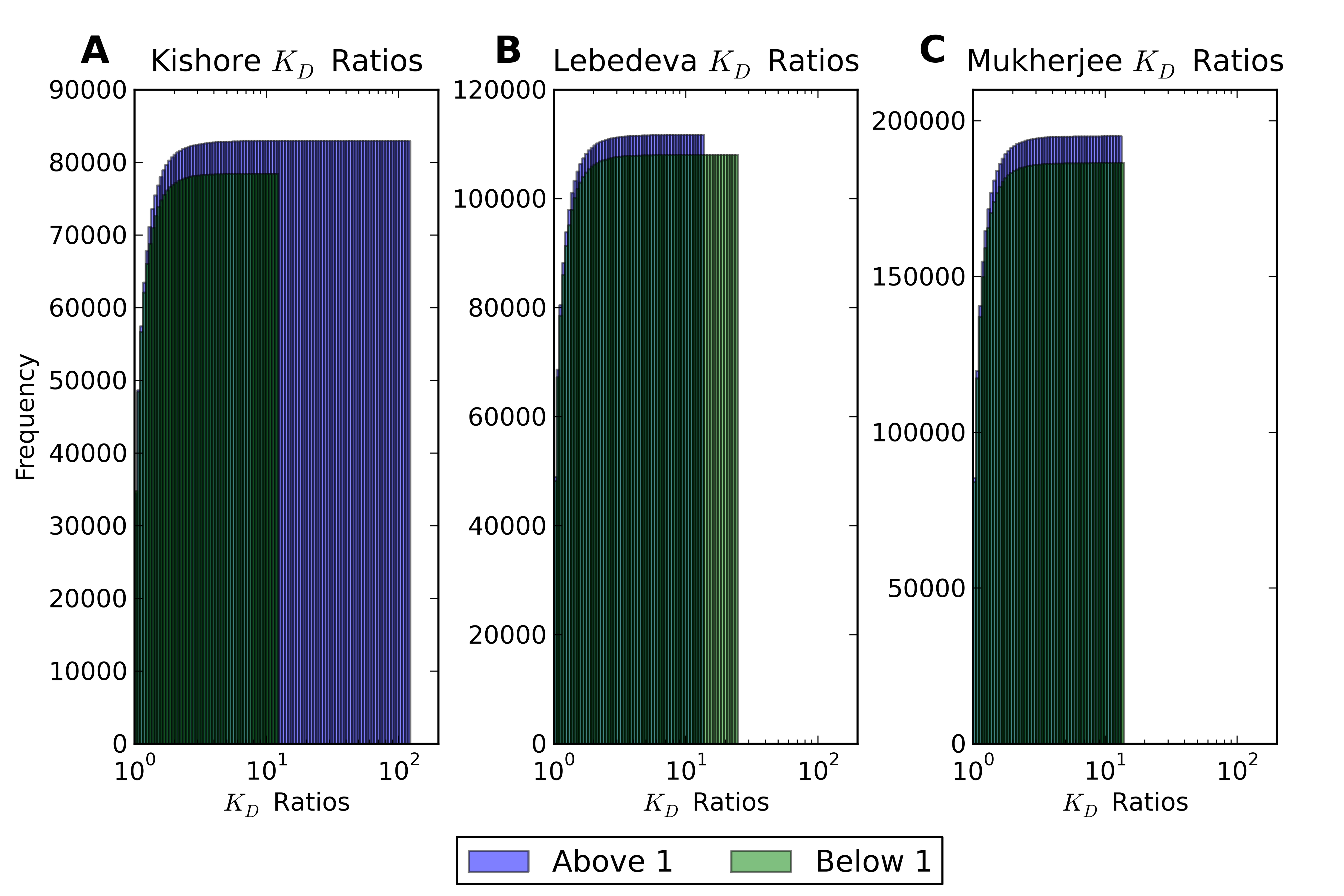

Supplement: S12 Fig — (TIF) [file pcbi.1007852.s014.tif]
